# Efficacy of different acupuncture-related therapies for tension-type headache: a systematic review and network meta-analysis

**DOI:** 10.3389/fneur.2024.1481715

**Published:** 2024-12-05

**Authors:** Yimeng Wang, Wang Lu, Yongjun Wang, Wei Chen, Haiyin Zhao

**Affiliations:** ^1^Department of Acupuncture, Longhua Hospital Affiliated to Shanghai University of Traditional Chinese Medicine, Shanghai, China; ^2^Key Laboratory of Theory and Therapy of Muscles and Bones, Shanghai University of Traditional Chinese Medicine, Shanghai, China; ^3^Department of Pharmacy, Emergency General Hospital, Beijing, China

**Keywords:** acupuncture, tension-type headache, systematic review, network meta-analysis, clinical efficacy

## Abstract

**Background:**

Tension-type headache (TTH) is among the most common primary headache disorders, characterized by recurrent episodes that are difficult to manage, thus posing a significant public health challenge. Acupuncture, a well-recognized non-pharmacological treatment, is frequently employed for pain management, including TTH. However, the variety of acupuncture techniques and inconsistent treatment outcomes underscore the need for a thorough evaluation. This study aims to update the current evidence on acupuncture and related therapies for TTH, evaluate the efficacy and safety of various acupuncture therapies, and identify the most effective therapeutic strategies, providing valuable guidance for clinical practice.

**Methods:**

We systematically searched randomized controlled trials (RCTs) from four English databases (PubMed, Embase, Cochrane Library, and Web of Science) and four Chinese databases (Wanfang, VIP, CNKI, and SinoMed), including gray literature, up to April 19, 2024. The outcome measures included headache frequency, duration, pain intensity, and responder rate. A Bayesian network meta-analysis was conducted using Stata 17.0 to assess the relative effectiveness and safety of the different acupuncture therapies. This study was registered with the Prospective Register of Systematic Reviews (CRD42024537187).

**Results:**

A total of 42 RCTs, encompassing 4,103 participants and 21 distinct treatment therapies, were included in the analysis. The network meta-analysis yielded the following findings: (1) regarding responder rate, several acupuncture or combined acupuncture and medication approaches, such as electro-acupuncture (EA) + cupping therapy (CT) [odds ratio (OR) = 28.66, 95% CI: 1.68 to 487.35], manual acupuncture (MA) + bloodletting therapy (BT) (OR = 6.07, 95% CI: 1.81 to 20.29), plum blossom needle tapping (PBNT) (OR = 3.76, 95% CI: 1.04 to 13.65), and scalp acupuncture (SPA) (OR = 3.65, 95% CI: 2.29 to 5.83), were significantly more effective than western medicine (WM) alone, with EA + CT (92.1%) being the most effective. (2) In terms of reducing headache frequency, EA (85.9%) was the most effective, followed by MA + PBNT (80.9%) and MA + WM (78.4%). Compared to WM, both MA + PBNT (SMD = −1.76, 95% CI: −3.31 to −0.22) and EA (SMD = −1.75, 95% CI: −3.30 to −0.20) significantly reduced headache frequency. (3) For shortening headache duration, EA (83.9%) emerged as the most effective treatment, followed by MA + WM (73.5%) and laser acupuncture (LA) (68.5%). (4) In terms of pain intensity reduction, the MA + WM combination (89.4%) was superior to other treatments, with SPA + WM (77.7%) being the next most effective. Compared to herbal medicine (HM), both MA + WM (SMD = −2.37, 95% CI: −4.20 to −0.55) and MA alone (SMD = −1.00, 95% CI: −1.75 to −0.24) significantly alleviated pain intensity.

**Conclusion:**

This comprehensive analysis of 21 acupuncture and related therapies demonstrates that EA is the most effective in reducing headache frequency and shortening headache duration, while EA + CT and MA + WM are the optimal therapies for enhancing responder rate and reducing pain intensity, respectively. However, clinical decisions should be individualized based on the specific needs of each patient.

**Systematic review registration:**

The study protocol was registered on the PROSPERO database under registration number CRD42024537187 (https://www.crd.york.ac.uk/prospero/#recordDetails).

## 1 Introduction

Tension-type headache (TTH) is a prevalent primary headache disorder, commonly encountered in clinical practice. It is typically characterized by mild to moderate tightening, pressing, or dull pain, often bilaterally located in the temporal regions and potentially extending to the forehead and occipital areas (1). TTH is marked by persistent, recurrent episodes that are challenging to manage effectively. This condition frequently leads to negative emotional states, including irritability, insomnia, anxiety, and depression, which can, in turn, exacerbate headache recurrence. This vicious cycle significantly impairs patients’ ability to learn and work, thereby diminishing their quality of life and contributing to substantial social and economic burdens (2, 3). The global incidence of TTH has remained consistently high in recent years. The Global Burden of Disease (GBD) study reported 882.4 million new cases of TTH worldwide in 2017 (4), with further research indicating that 26% of the global population experiences TTH annually (5).

Western medicine offers various treatment strategies tailored to different types of TTH (1, 6). For infrequent episodic TTH, symptomatic relief is typically achieved through the use of analgesics or non-steroidal anti-inflammatory drugs (NSAIDs) (7). In contrast, the management of chronic or frequent episodic TTH often involves preventive treatments, including tricyclic antidepressants such as amitriptyline and mirtazapine (7, 8), in combination with non-pharmacological interventions like physical exercise or cognitive behavioral therapy (9, 10). However, prolonged or excessive use of analgesics or NSAIDs may result in adverse effects, such as gastrointestinal ulcers and renal impairment (11). Furthermore, tricyclic antidepressants, including mirtazapine, are associated with side effects like urinary retention, constipation, and cognitive dysfunction (12, 13). These limitations highlight the need for more effective non-pharmacological treatments (7).

Acupuncture, a cornerstone of traditional medicine with a history spanning thousands of years, has been extensively studied and recognized for its significant efficacy in pain management (14). The analgesic effects of acupuncture are attributed to mechanisms such as the modulation of the central and peripheral nervous systems (15), the promotion of endogenous opioid peptide release (16), and the regulation of cytokine and other immune-active substances (17). Due to its potent pain-relieving effects and minimal side effects, acupuncture has been widely adopted in the treatment of TTH (14, 18). Various acupuncture techniques are used in TTH management, including manual acupuncture, electroacupuncture, scalp acupuncture, and bloodletting therapy (19–21).

However, the absence of direct head-to-head comparisons among different acupuncture therapies leaves the optimal treatment for TTH undetermined. This study seeks to evaluate the safety and efficacy of various acupuncture therapies through a Bayesian network meta-analysis, employing indirect comparisons to provide evidence-based support for the clinical management of TTH.

## 2 Methods

### 2.1 Registration

This study was conducted in accordance with the AMSTAR2 guidelines (A Measurement Tool to Assess Systematic Reviews) and reported in line with the PRISMA (Preferred Reporting Items for Systematic Reviews and Meta-Analyses) guidelines specific to Network Meta-Analyses (Supplementary Table S1). The study protocol was registered on the PROSPERO database under registration number CRD42024537187.^1^

### 2.2 Search strategy

We performed a comprehensive literature search across eight databases: four English-language databases (PubMed, Embase, Cochrane Library, and Web of Science) and four Chinese-language databases (Wanfang, VIP, CNKI, and SinoMed). To ensure the inclusion of all relevant studies, we manually searched the reference lists of the included articles and related reviews. Gray literature, such as theses and conference proceedings, was also reviewed. There were no restrictions on publication date or language. The search utilized terms related to acupuncture (e.g., “acupuncture,” “electroacupuncture,” “manual acupuncture,” “moxibustion,” “scalp acupuncture,” “auricular acupuncture”), TTH (e.g., “tension-type headache,” “vascular tension headache,” “idiopathic headache,” “headache”), and study design (e.g., “randomized controlled trial” or “clinical trial”). For the Chinese databases, equivalent Chinese terms were employed. The complete search strategy is detailed in Supplementary Table S2.

References retrieved from the databases were managed using EndNote (version 20) software. The following study characteristics were summarized in a Microsoft excel spreadsheet: publication year, author, population, intervention type, outcome measures, control group, and demographic details (e.g., gender, age).

### 2.3 Eligibility criteria

#### 2.3.1 Inclusion criteria

Study design: RCTs.

Participants: Patients diagnosed with TTH based on the International Headache Society (IHS) criteria (22). A detailed review of the studies published before 2018 was conducted, focusing specifically on key factors like headache frequency, duration, and symptom characteristics, to assess compatibility with the IHS 2018 criteria. Studies that did not meet the specific IHS 2018 standards were excluded to maintain diagnostic uniformity.

Interventions: Any form of acupuncture, including body acupuncture, scalp acupuncture, auricular acupuncture, or electroacupuncture.

Control groups: Studies where the control group received an alternative form of acupuncture or related therapy, including sham acupuncture, usual care, waitlist, or other active treatments (e.g., guideline-recommended pharmacotherapy, physical exercise, relaxation training, cognitive therapy).

Outcomes: The primary outcome was responder rate, and the secondary outcomes were headache frequency, headache duration, pain intensity and adverse reactions.

Publication types: Journal articles and theses.

Data requirements: Studies must include original data on relevant outcomes, or data that could be extracted from figures and tables.

#### 2.3.2 Exclusion criteria

- Studies where the intervention group received additional treatments beyond acupuncture or related therapies.
- Duplicate data.
- Animal studies.
- Studies published solely as conference abstracts, dissertations, study protocols, or books.

### 2.4 Outcome measurements

#### 2.4.1 Primary outcome

Responder rate: A participant with a > 50% reduction in monthly headache days after treatment (23).

#### 2.4.2 Secondary outcome

Headache frequency: Number of headache days within the specified period.

Headache duration: Lasting hours of headache within the specified period.

Pain intensity: Visual Analog Scale (VAS).

Adverse reactions: Subcutaneous hematoma, pain in acupuncture site, temporary headache triggered by needling, etc.

### 2.5 Data extraction

Two independent reviewers extracted data from the eligible studies, capturing details on publication (author, year of publication, country), study characteristics (sample size, intervention type), participant characteristics (sample size, age, gender), and study design (randomized controlled trials). The primary outcomes of interest were responder rate, headache frequency, headache duration, and pain intensity.

Outcome data before and after the intervention (mean ± standard deviation) were extracted and summarized to evaluate the effectiveness of various acupuncture techniques in treating TTH. When studies reported standard errors (SE), confidence intervals (CIs), or interquartile ranges (IQRs) instead of means and standard deviations, these were converted to mean and standard deviation values using the RevMan 5.3 calculator.

### 2.6 Quality and risk of bias assessment

Two independent reviewers assessed the risk of bias in the included studies using the Cochrane Risk of Bias tool (ROB 2.0), evaluating 6 domains, including randomization process, deviations from intended interventions, missing outcome data, measurement of the outcome, selection of the reported results, and overall evalution. This tool categorizes risk into three levels: low, high, and unclear. Given the nature of interventions, blinding of participants and personnel were generally considered high risk. Discrepancies between reviewers were resolved through discussion with the corresponding author to reach consensus.

### 2.7 Data analysis

In this study, we performed a Bayesian network meta-analysis using STATA 17.0. To begin, a network evidence diagram was created, where node size indicates the number of studies for each intervention, and line thickness between nodes represents the frequency of comparisons between intervention pairs. For networks containing closed loops, a global inconsistency test was conducted with a significance level of *α* = 0.05. A *p*-value of <0.05 suggests inconsistency, indicating the need for sensitivity analysis to examine potential heterogeneity. In the absence of closed loops or if no significant inconsistency is observed (*p* > 0.05), a consistency model is applied to the network meta-analysis, followed by the creation of a forest plot. Additionally, we utilized the node-splitting method to assess local inconsistencies. A *p*-value <0.05 in this context suggests local inconsistency, requiring careful interpretation of these results.

Additionally, to ensure the model alignment in our analysis, we performed model comparison using the deviance information criterion (DIC). This metric evaluates the relative fit of fixed-effect and random-effect models, with lower DIC values indicating better model adequacy. Consistency between fixed-effect and random-effect models is confirmed when the DIC difference is less than 5. This approach allowed us to select the most appropriate model for each cohort, enhancing the precision of our analysis.

For effect size determination, continuous variables such as headache frequency, duration, and pain intensity are assessed using the mean difference (MD) when outcome units are consistent. For continuous variables with non-uniform units, the standardized mean difference (SMD) is employed to account for variability. The responder rate, a binary variable, is measured using the OR, with a 95% CI calculated for analytical accuracy. Results of the network meta-analysis are displayed in a league table format, providing a comparative ranking of each intervention. Additionally, we construct a surface under the cumulative ranking curve (SUCRA) to represent the efficacy rankings of interventions, with SUCRA values ranging from 0 to 100%. Higher SUCRA values reflect greater effectiveness, while lower values suggest less efficacy. Finally, we generate a comparison-adjusted funnel plot to detect potential publication bias and small-study effects.

## 3 Results

### 3.1 Search results

A comprehensive literature search was conducted across eight databases, covering all publications up to April 2024. This search identified 2,442 studies from English-language databases (PubMed, Web of Science, Cochrane Library, Embase) and 8,724 studies from Chinese-language databases (Wanfang, CNKI, VIP, SinoMed). After removing 5,638 duplicate records using EndNote, 5,458 studies were excluded based on title and abstract screening.

**Figure 1:**
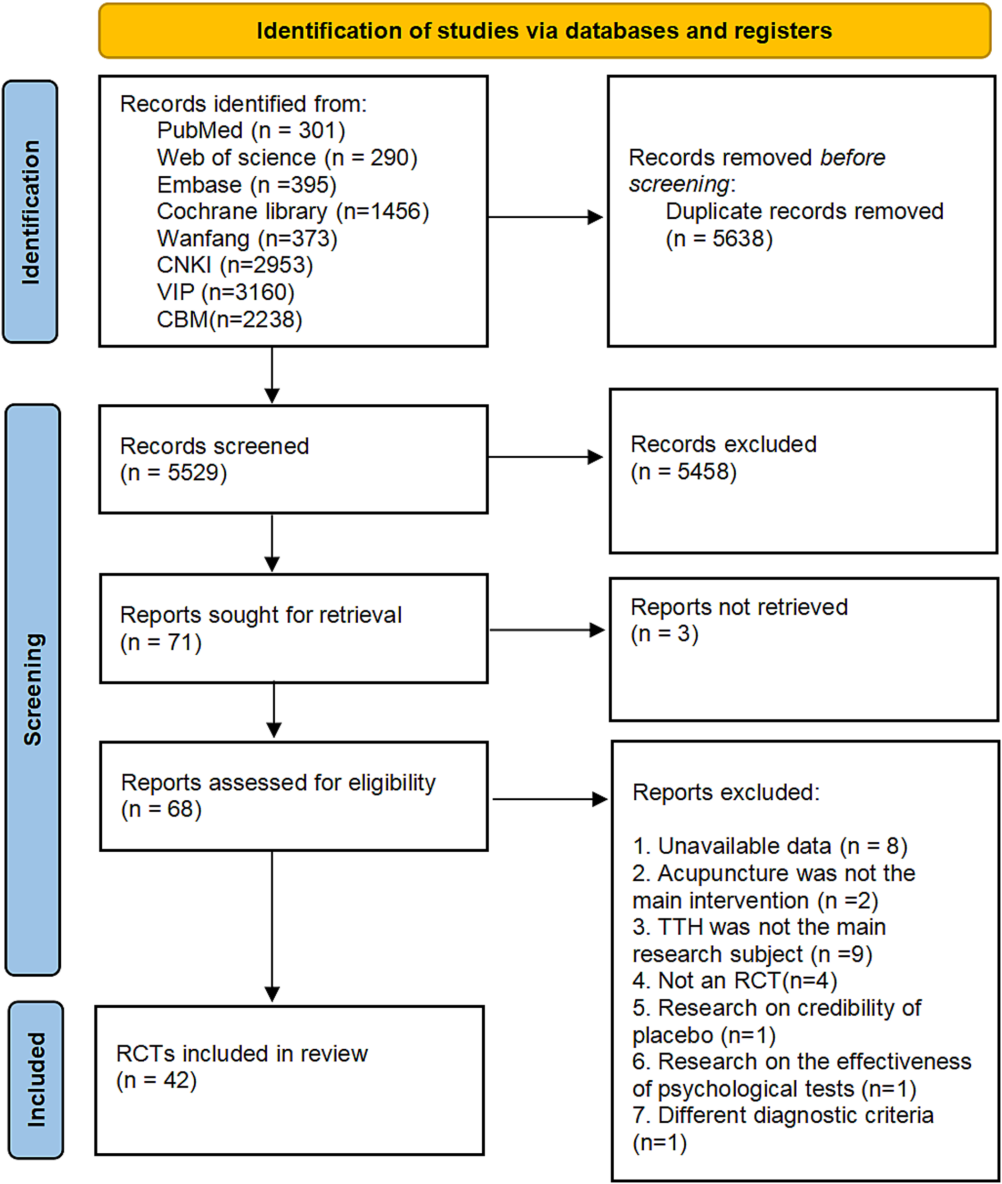
Literature screening process.

The full texts of the remaining 71 studies were retrieved for detailed evaluation, but three studies were excluded due to the unavailability of their full text. Of the 68 studies assessed in full, 26 were excluded for the following reasons: insufficient data (*n* = 8), acupuncture not being the primary intervention (*n* = 2), TTH not being the primary focus (*n* = 9), non-randomized controlled trial design (*n* = 4), focus on placebo effects (*n* = 1), focus on psychological test efficacy (*n* = 1), and use of non-standard diagnostic criteria (*n* = 1), and the details for exclusion are listed in Supplementary Table S3. Consequently, 41 studies were included in the final analysis. The study selection process is depicted in Figure 1.

### 3.2 Characteristics of included studies

Table 1 presents a summary of the key characteristics of the 42 randomized controlled trials (RCTs) included in this analysis. The sample sizes of these studies ranged from 30 to 270 participants, with the average age of participants spanning from 30 to 51 years. All studies included both male and female participants. The research teams were based in nine different countries: China (*n* = 30), Germany (*n* = 5), Sweden (*n* = 1), Australia (*n* = 1), South Korea (*n* = 1), Iran (*n* = 1), Brazil (*n* = 1), Denmark (*n* = 1), and the United Kingdom (*n* = 1).

**Table 1:** Characteristics of the included studied.

References	Country	Intervention				Comparison				Outcome
		Treatment	Sample size	Gender (male/female)	Age (mean ± SD)/range	Treatment	Sample size	Gender (male/female)	Age (mean ± SD)/range	
Zheng et al. (31)	China	MA	110	28/82	43 ± 12.5	SA	108	33/75	43.2 ± 12.8	① ② ④ ⑤
						UC	24	7/17	38.7 ± 14.6	
Schiller et al. (32)	Germany	MA	24	6/18	39.8 ± 12.2	PT	24	5/18	37 ± 15.3	① ② ③ ⑤

						MA + PT	24	2/22	39 ± 11.6	
Jeon and Lee (33)	Korea	EA	15	6/9	40.00 ± 13.11	SA	15	8/7	34.33 ± 11.48	② ④
Chassot et al. (34)	Brazil	EA	18	0/18	39.11 ± 10.5	SA	16	0/16	41.44 ± 10.5	④
Endres et al. (25)	Germany	MA	209	46/163	39.2 ± 11.4	SA	200	42/158	38.9 ± 12.2	① ② ⑤
Wang et al. (35)	Denmark	EA	20	8/10	43.24 ± 6.44	SA	20	10/8	53.39 ± 5.80	② ③ ④
Söderberg et al. (36)	Sweden	MA	30	7/23	35 ± 10.25	PT	3030	7/23	35 ± 9.5	② ③ ④
						RT	30	3/27	43.5 ± 9.25	
Melchart et al. (37)	Germany	MA	132	72/95	42.3 ± 13.5	SA	63	73/46	43.4 ± 12.9	② ③
						WL	75	77/58	42.8 ± 13.2	
Ebneshahidi et al. (38)	Iran	LA	25	6/19	33 ± 6.75	SA	25	6/19	38.6 ± 7	② ③
Xue et al. (39)	Australia	EA	20	7/13	42.6 ± 1.8	SA	20	7/13	41.5 ± 1.9	② ③ ④
Karst et al. (40)	Germany	MA	34	14/14	47.9 ± 13.8	SA	35	14/21	48.2 ± 14.6	② ④
White et al. (41)	England	MA	25	7/18	49.8 ± 2.9	SA	25	5/20	48.2 ± 2.9	② ③ ④ ⑤
Karst et al. (42)	Germany	MA	21	13/8	50.4 ± 13.5	SA	18	7/11	47.3 ± 16.5	② ④
Tong et al. (43)	China	MA + HM	43	16/27	43.4 ± 11.2	WM	43	14/29	42.7 ± 10.4	① ② ③ ④
Shi (44)	China	MA + HM	45	19/26	36 ± 10.5	WM	45	16/29	41 ± 8.5	①
Zhou (45)	China	MA + WM	34	22/12	48.21 ± 3.80	WM	34	20/14	47.62 ± 3.11	① ② ③ ④
Zhang et al. (46)	China	MA	26	NR	41.5 ± 11.75	SA + WM	20	NR	41.5 ± 11.75	① ② ③
Peng (47)	China	MA	63	28/35	32.5 ± 5.6	WM	63	26/37	31.3 ± 6.3	①
Li and Liu (48)	China	MA	40	NR	39 ± 10.5	HM	40	NR	39 ± 10.5	① ④
Chen et al. (49)	China	MA	34	9/25	32.6 ± 4.5	HM	32	15/17	37.2 ± 6.2	①
Duan (50)	China	MA	48	14/34	42.7 ± 11.5	WM	48	17/31	43.5 ± 11.2	③ ④
Chen and Feng (51)	China	MA	70	22/48	39.89 ± 9.48	WM	70	18/52	40.36 ± 9.48	①
Guo (52)	China	MA + PBNT	30	13/17	26.5 ± 15.2	WM	30	12/17	24.6 ± 15.4	② ③
Zou et al. (53)	China	MA + BT	30	11/19	36.54 ± 11.60	MA	30	13/17	37.93 ± 11.80	①
						HM	30	16/14	39.23 ± 12.24	
Guo (54)	China	MA + HM	40	25/15	36.5 ± 3.2	WM	40	21/19	38.7 ± 3.8	① ④
Wang et al. (55)	China	MA	50	27/23	37.64 ± 3.42	WM	50	23/26	35.17 ± 4.03	② ③ ④
Kang (56)	China	MA + HM	58	20/38	33.5 ± 7.25	HM	33	12/21	30 ± 5	①
Zhang (57)	China	MA + HM	38	16/22	39.3 ± 5.1	WM	38	15/23	39.7 ± 5.2	①
Wu and Sun (58)	China	MA + HM	30	13/17	39 ± 10.5	WM	30	14/16	39 ± 11	①
Gui et al. (59)	China	SPA + WM	30	13/17	38 ± 13	WM	30	19/11	39 ± 11	①
Li et al. (60)	China	MA	30	NR	40 ± 10	WM	25	NR	40 ± 10	①
Jiang (61)	China	MA + HM	40	17/23	39.7 ± 4.3	WM	40	18/22	38.5 ± 4.8	①
Luo et al. (62)	China	MA	42	17/25	45.31 ± 7.58	WM	43	16/27	45.13 ± 8.12	①
Liu and Liu (63)	China	MA + HM	91	68/23	42 ± 12	HM	31	12/19	41.5 ± 11.25	①
Yang et al. (64)	China	MA	41	31/10	40.96 ± 8.23	WM	41	33/8	41.29 ± 8.32	①
Wang et al. (65)	China	MA	150	63/87	44 ± 13	WM	100	42/58	43 ± 13	① ③
Chen et al. (49)	China	BT	45	20/25	44 ± 11.5	MA	45	18/27	41 ± 11.5	④
Sun and Guo (66)	China	PBNT	30	11/19	43.5 ± 11.7	WM	30	9/21	42.8 ± 11.3	① ② ③ ④
Peng (67)	China	EA + CT	82	38/44	36.23 ± 6.25	WM	81	36/45	34.68 ± 5.27	①
Jin et al. (68)	China	BT	30	12/18	45.3 ± 3.9	WM	30	14/16	43.2 ± 3.2	④
Guo and Shen (69)	China	SPA	50	22/28	33.9 ± 10.2	MA	50	16/34	33.2 ± 10.2	①
						WM	50	19/31	34.0 ± 10.6	
Wang et al. (70)	China	MA	29	8/21	38 ± 10	WM	27	2/20	39 ± 11	① ② ③ ④

①, Responder rate (at least 50% reduction of headache days); ②, Headache frequency; ③, Headache duration; ④, Pain intensity; ⑤, Adverse reaction; MA, manual acupuncture; SA, sham acupuncture; PT, physical training; UC, usual care; HM, herbal medicine; WM, western medicine; BT, bloodletting therapy; SPA, scalp acupuncture; PBNT, plum blossom needle tapping; EA, electro-acupuncture; CT, cupping therapy; RT, relaxation training; WL, waiting list; LA, laser acupuncture.

Among the studies, one trial featured four participant groups, four trials included three groups, and the remaining studies were two-group trials. The interventions in the treatment groups varied, including manual acupuncture (*k* = 20), acupuncture combined with herbal medicine (*k* = 8), electroacupuncture (*k* = 4), bloodletting therapy (*k* = 2), acupuncture combined with bloodletting therapy (*k* = 1), scalp acupuncture (*k* = 1), electroacupuncture combined with cupping therapy (*k* = 1), laser acupuncture (*k* = 1), plum-blossom needle therapy (*k* = 1), acupuncture combined with plum-blossom needle therapy (*k* = 1), acupuncture combined with western medication (*k* = 1), and scalp acupuncture combined with western medication (*k* = 1).

The control groups were subjected to various treatments, including western medication (*k* = 22), sham acupuncture (*k* = 11), herbal medicine (*k* = 5), physical exercise (*k* = 2), relaxation training (*k* = 1), sham acupuncture combined with western medication (*k* = 1), usual care (*k* = 1), waiting list (*k* = 1), and acupuncture combined with physical exercise (*k* = 1).

### 3.3 Risk of bias assessment

**Figure 2:**
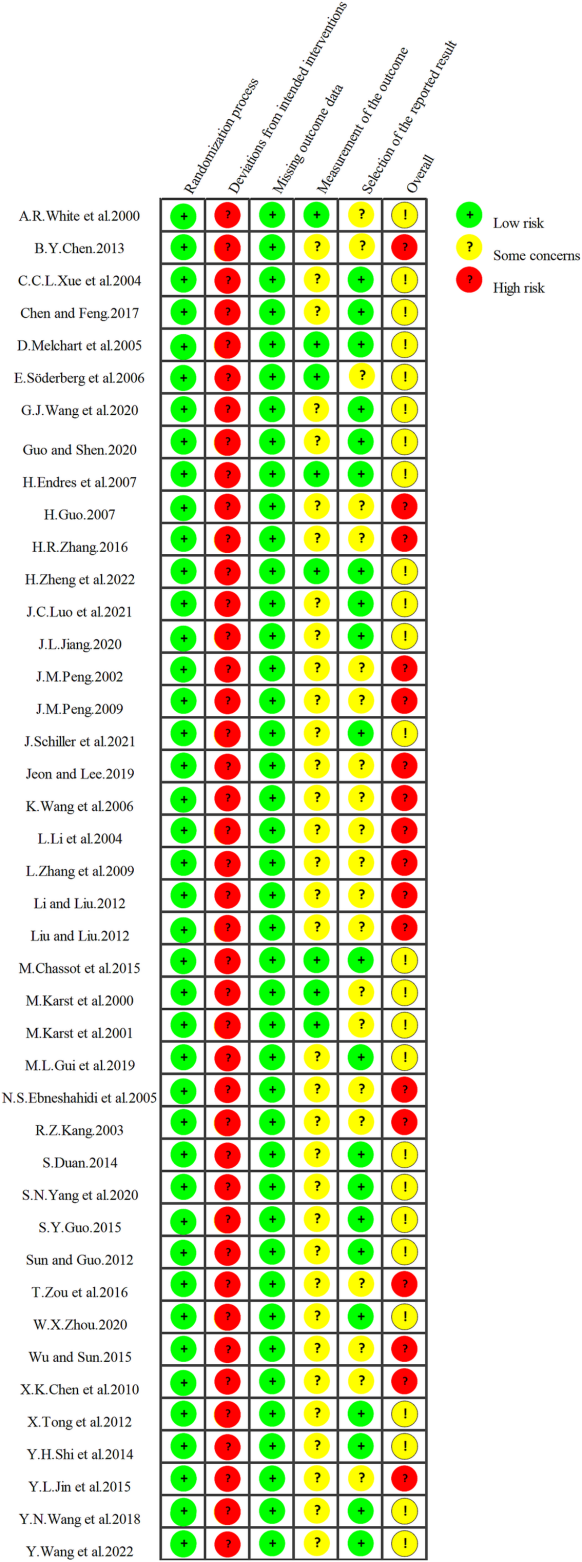
Results of the risk of bias evaluation.

In this study, all 42 included trials reported a randomization process. Each study was rated as high risk for bias related to deviations from intended interventions, primarily due to the lack of blinding for both participants and personnel. Missing outcome data bias was consistently assessed as low risk, as dropout numbers and reasons were clearly reported. Outcome assessment bias was rated as low in 8 studies where blinding of outcome assessors was documented; for the remaining 34 studies, the lack of reported outcome assessor blinding led to the some concerns rating. Reporting bias was determined based on whether all anticipated outcomes were reported, with 50% of the studies rated as low risk and the other half as having some concerns. In summary, 17 studies were rated as high risk for overall bias, while the remaining 25 were assessed as having some concerns. Detailed risk of bias assessments for all included studies are illustrated in Figure 2.

### 3.4 Network meta-analysis results

#### 3.4.1 Headache frequency

**Figure 3:**
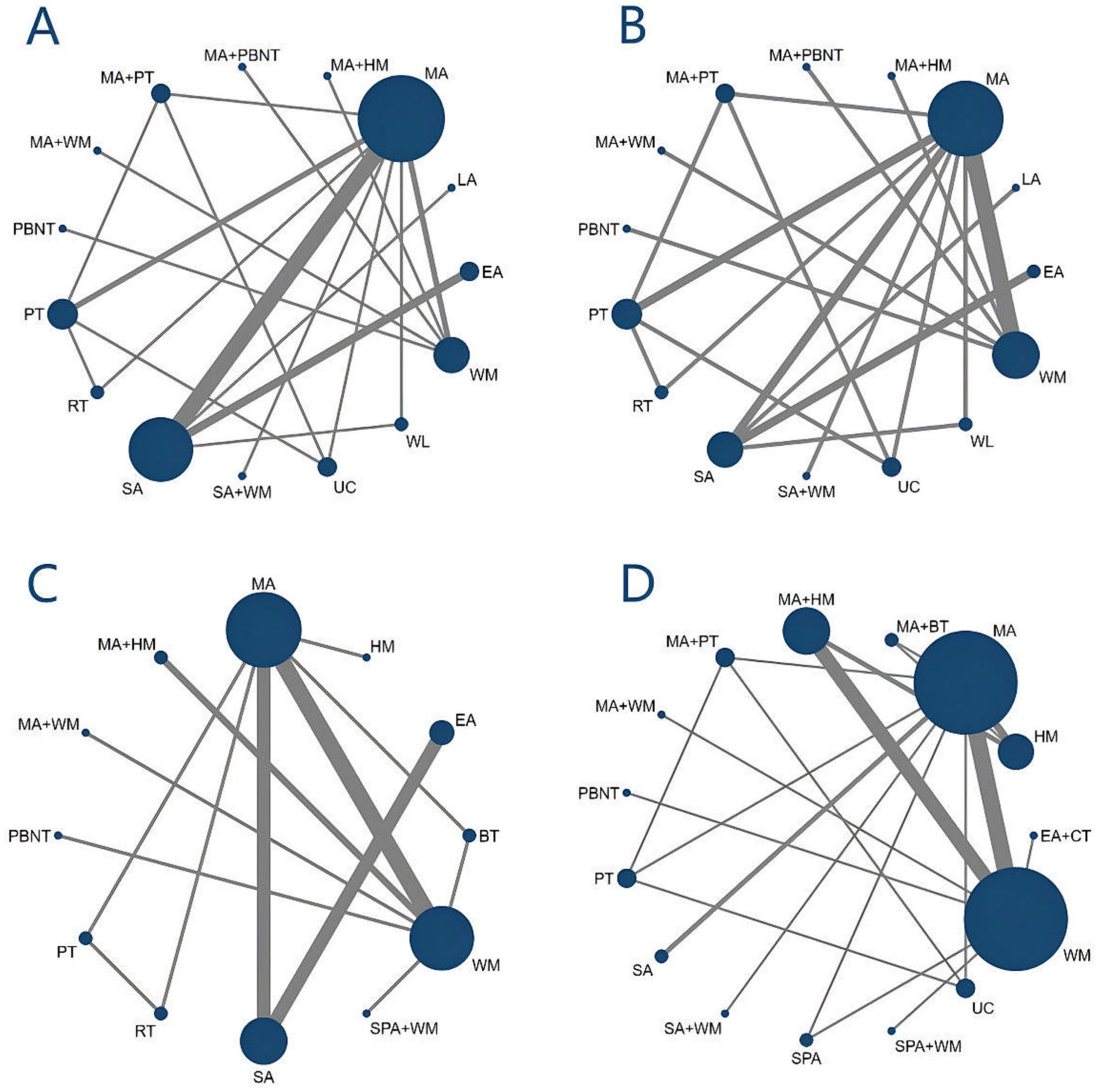
Evidence network diagram. **(A)** Headache frequency. **(B)** Headache duration. **(C)** Pain intensity. **(D)** Responder rate.

Nineteen studies addressing headache frequency were analyzed, encompassing 15 different treatment modalities. The network diagram of these treatments is presented in Figure 3A. The network meta-analysis revealed a closed-loop structure. The inconsistency test for this closed loop indicated *p* ≥ 0.05, suggesting no significant evidence of inconsistency; therefore, a consistency model was utilized for the analysis.

**Figure 4:**
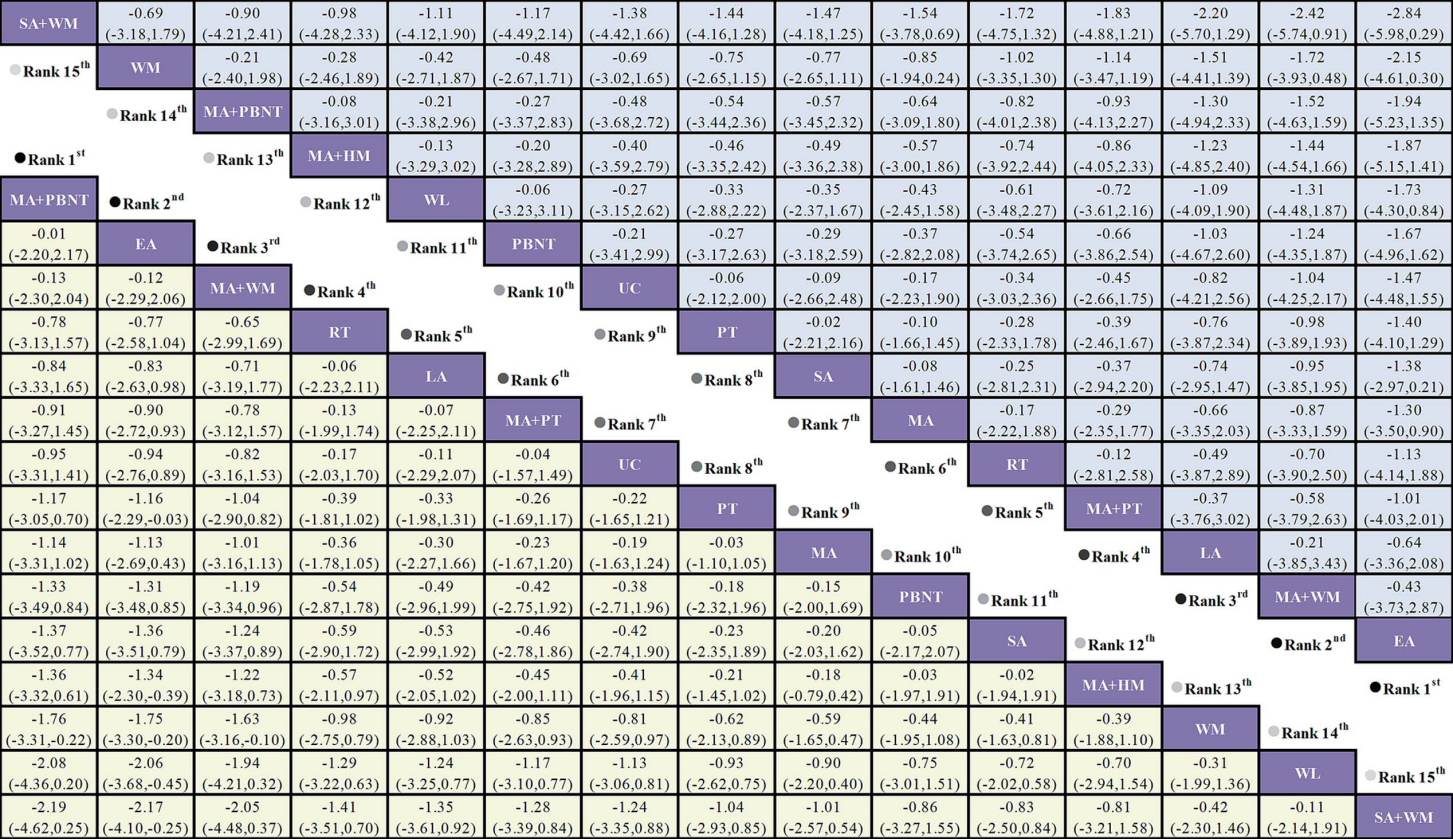
Forest plot comparing the efficacy of various acupuncture treatments in headache frequency and headache duration of TTH based on Bayesian network meta-analysis. SMD and 95% CIs for headache frequency (depicted by the yellow lower triangle area) and headache duration (depicted by the blue upper triangle area). 95% CIs <0.00 indicates a statistically significant difference between two treatments.

According to the NMA results, both MA (manual acupuncture) + PBNT (plum blossom needle tapping) (SMD = −1.76, 95% CI: −3.31 to −0.22) and EA (electro-acupuncture) (SMD = −1.75, 95% CI: −3.30 to −0.20) significantly reduced headache frequency compared to WM (western medicine). EA demonstrated a significant reduction in headache frequency compared to PT (physical training) (SMD = −1.16, 95% CI: −2.29 to −0.03), MA + HM (herbal medicine) (SMD = −1.34, 95% CI: −2.30 to −0.39), WL (waiting list) (SMD = −2.06, 95% CI: −3.68 to −0.45), and SA (sham acupuncture) + WM (SMD = −2.17, 95% CI: −4.10 to −0.25). No significant differences were observed between the remaining treatment comparisons, as illustrated in Figure 4.

**Figure 5:**
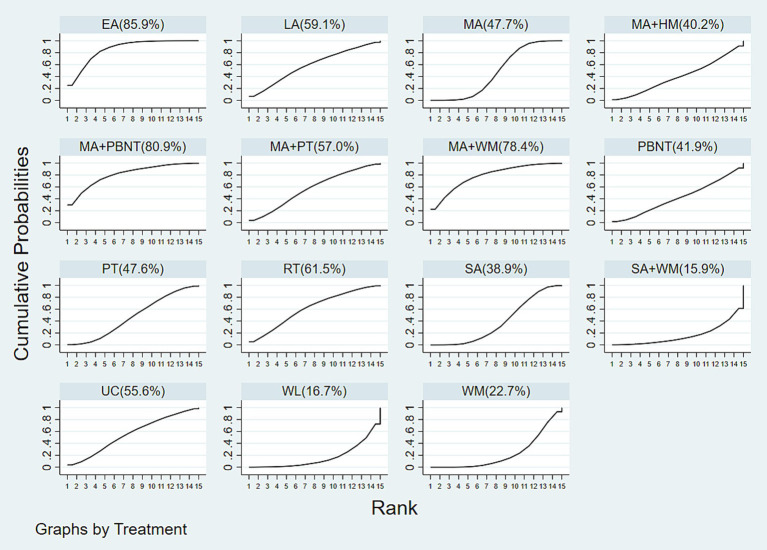
Cumulative probability ranking results of headache frequency.

The cumulative probability ranking for effectiveness in reducing headache frequency is as follows: EA (85.9%) > MA + PBNT (80.9%) > MA + WM (78.4%) > RT (relaxation training) (61.5%) > LA (laser acupuncture) (59.1%) > MA + PT (57.0%) > UC (usual care) (55.6%) > MA (47.7%) > PT (47.6%) > PBNT (41.9%) > MA + HM (40.2%) > SA (38.9%) > WM (22.7%) > WL (16.7%) > SA + WM (15.9%), as depicted in Figure 5. The funnel plot analysis, conducted using Stata 17.0 and included in the Supplementary Figure S1, reveals some asymmetry, which may suggest potential publication bias or small-study effects within the research network.

#### 3.4.2 Headache duration

Sixteen studies investigated headache duration across 15 different treatments. The network diagram illustrating these treatments is shown in Figure 3B. The network meta-analysis revealed a closed loop in the network diagram. The inconsistency test for the closed loop yielded *p* ≥ 0.05, indicating no significant inconsistency. Therefore, a consistency model was utilized for the analysis. The NMA results indicated no statistically significant differences among the various treatments, suggesting that no single treatment was more effective than the others in reducing headache duration, see Figure 4.

**Figure 6:**
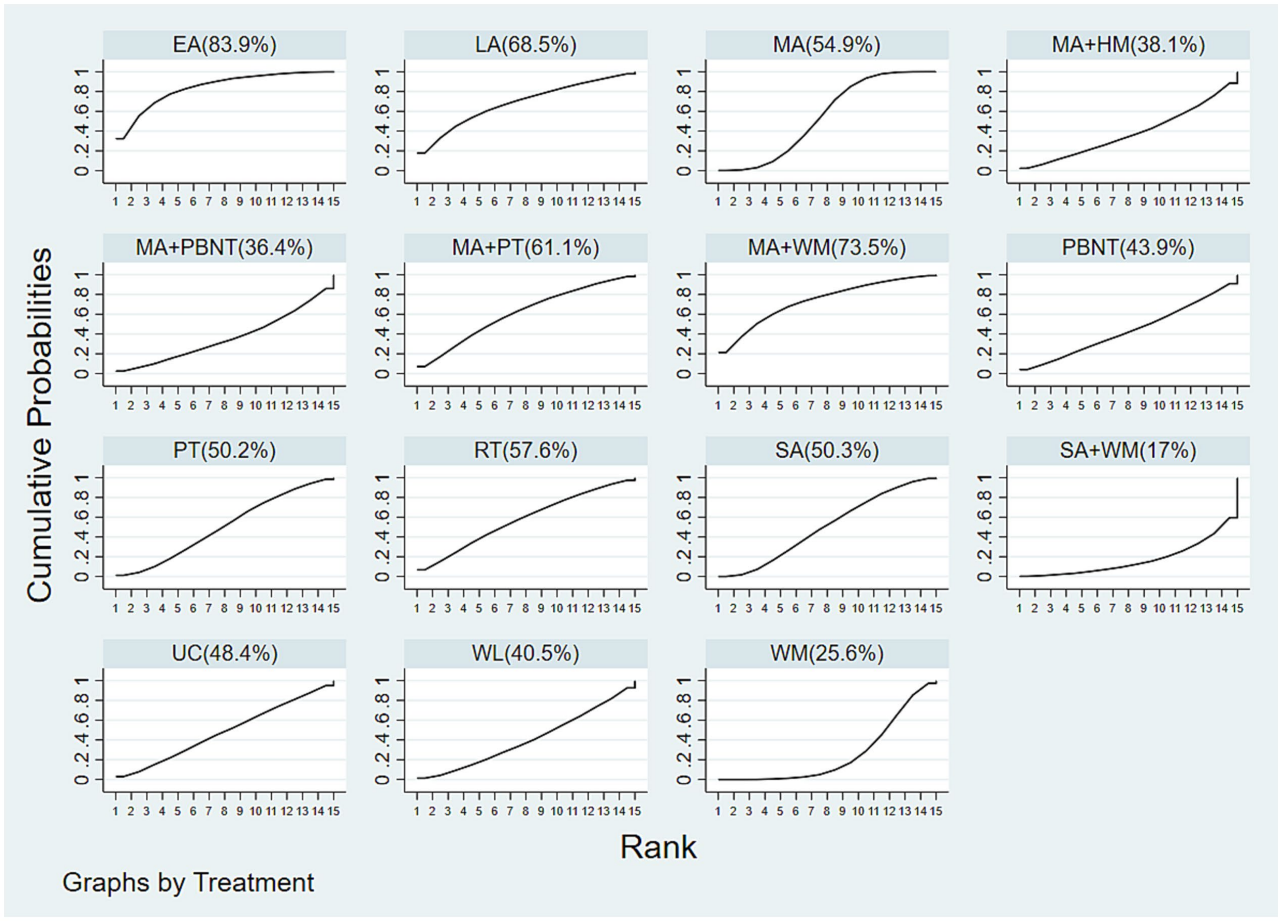
Cumulative probability ranking results of headache duration.

The cumulative probability rankings for reducing headache duration are as follows: EA (83.9%) > MA + WM (73.5%) > LA (68.5%) > MA + PT (61.1%) > RT (57.6%) > MA (54.9%) > SA (50.3%) > PT (50.2%) > UC (48.4%) > PBNT (43.9%) > WL (40.5%) > MA + HM (38.1%) > MA + PBNT (36.4%) > WM (25.6%) > SA + WM (17%), as depicted in Figure 6.

Funnel plot analysis, conducted using Stata 17.0 and provided in the Supplementary Figure S2, reveals that the funnel plot is not perfectly symmetrical. This suggests potential publication bias or small study effects within the research network.

#### 3.4.3 Pain intensity

**Figure 7:**
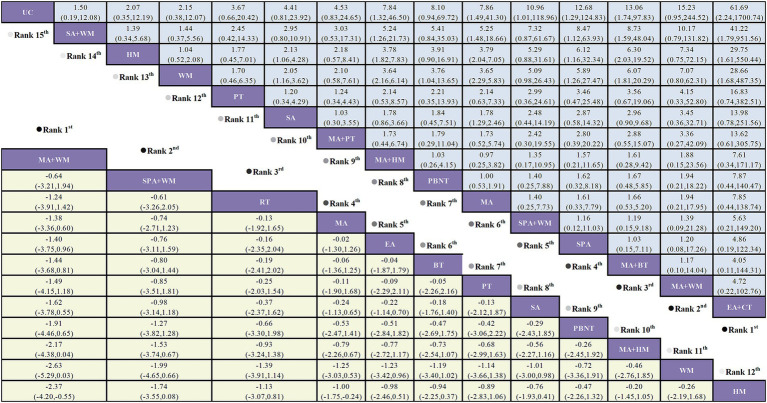
Forest plot comparing the efficacy of various acupuncture treatments in pain intensity and responder rate of TTH based on Bayesian network meta-analysis. SMD and 95% CIs for pain intensity (depicted by the yellow lower triangle area). 95% CIs <0.00 indicates a statistically significant difference between two treatments; OR and 95% CIs for responder rate (depicted by the blue upper triangle area). 95% CIs <1.00 or >1.00 indicates a statistically significant difference between two treatments.

A total of 22 studies examined pain intensity, involving 12 distinct treatment methods. The network diagram for these treatments is shown in Figure 3C. The network meta-analysis revealed a closed loop within this network. The inconsistency test for the closed loop yielded *p* ≥ 0.05, indicating no evidence of inconsistency; thus, a consistency model was employed for the analysis. According to the league table results, both MA + WM (SMD = −2.37, 95% CI: −4.20 to −0.55) and MA (SMD = −1.00, 95% CI: −1.75 to −0.24) significantly reduced pain intensity compared to HM. There were no statistically significant differences observed between other treatment comparisons, as detailed in Figure 7.

**Figure 8:**
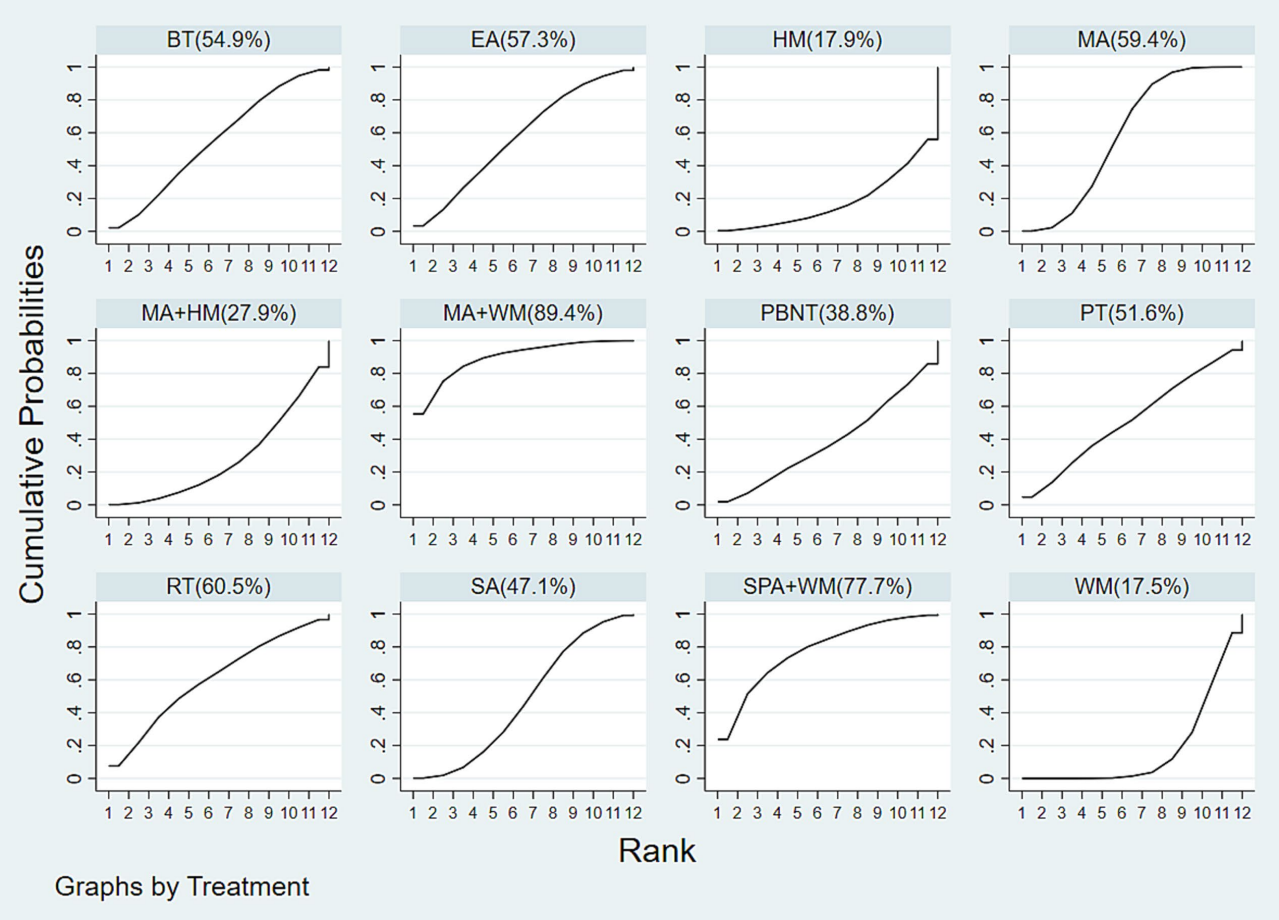
Cumulative probability ranking results of pain intensity.

The cumulative probability rankings for reducing pain intensity are as follows: MA + WM (89.4%) > SPA + WM (77.7%) > RT (60.5%) > MA (59.4%) > EA (57.3%) > BT (54.9%) > PT (51.6%) > SA (47.1%) > PBNT (38.8%) > MA + HM (27.9%) > HM (17.9%) > WM (17.5%), as shown in Figure 8.

Funnel plot analysis performed with Stata 17.0 is included in the Supplementary Figure S3. The results suggest that the funnel plot is not perfectly symmetrical, which may indicate potential publication bias or small study effects within the research network. The observed absence of studies with large sample sizes, as indicated by the missing top portion of the funnel plot, could suggest a lack of reporting or bias in the results from these studies.

#### 3.4.4 Responder rate

Twenty-six studies assessed the responder rate across 15 distinct treatment methods. The network diagram for these treatments is depicted in Figure 3D. The network meta-analysis revealed a closed loop within the network diagram. The inconsistency test for this closed loop yielded *p* ≥ 0.05, indicating no significant inconsistency; thus, a consistency model was applied for the analysis.

The NMA results showed that EA + CT was superior to SA + WM (OR = 41.22, 95% CI: 1.79 to 951.56), HM (OR = 29.75, 95% CI: 1.61 to 550.44), and WM (OR = 28.66, 95% CI: 1.68 to 487.35). MA + BT outperformed SA + WM (OR = 8.73, 95% CI: 1.59 to 48.04), HM (OR = 6.30, 95% CI: 2.03 to 19.52), and WM (OR = 6.07, 95% CI: 1.81 to 20.29). SPA was more effective than SA + WM (OR = 8.47, 95% CI: 1.12 to 63.93), HM (OR = 6.12, 95% CI: 1.16 to 32.34), and WM (OR = 5.89, 95% CI: 1.26 to 27.47). MA was superior to SA + WM (OR = 5.25, 95% CI: 1.48 to 18.66), HM (OR = 3.79, 95% CI: 2.04 to 7.05), and WM (OR = 3.65, 95% CI: 2.29 to 5.83). PBNT was more effective than WM (OR = 3.76, 95% CI: 1.04 to 13.65). MA + HM was more effective than SA + WM (OR = 5.24, 95% CI: 1.26 to 21.73), HM (OR = 3.78, 95% CI: 1.82 to 7.83), and WM (OR = 3.64, 95% CI: 2.16 to 6.14). No significant differences were observed between the remaining treatment comparisons, as illustrated in Figure 7.

**Figure 9:**
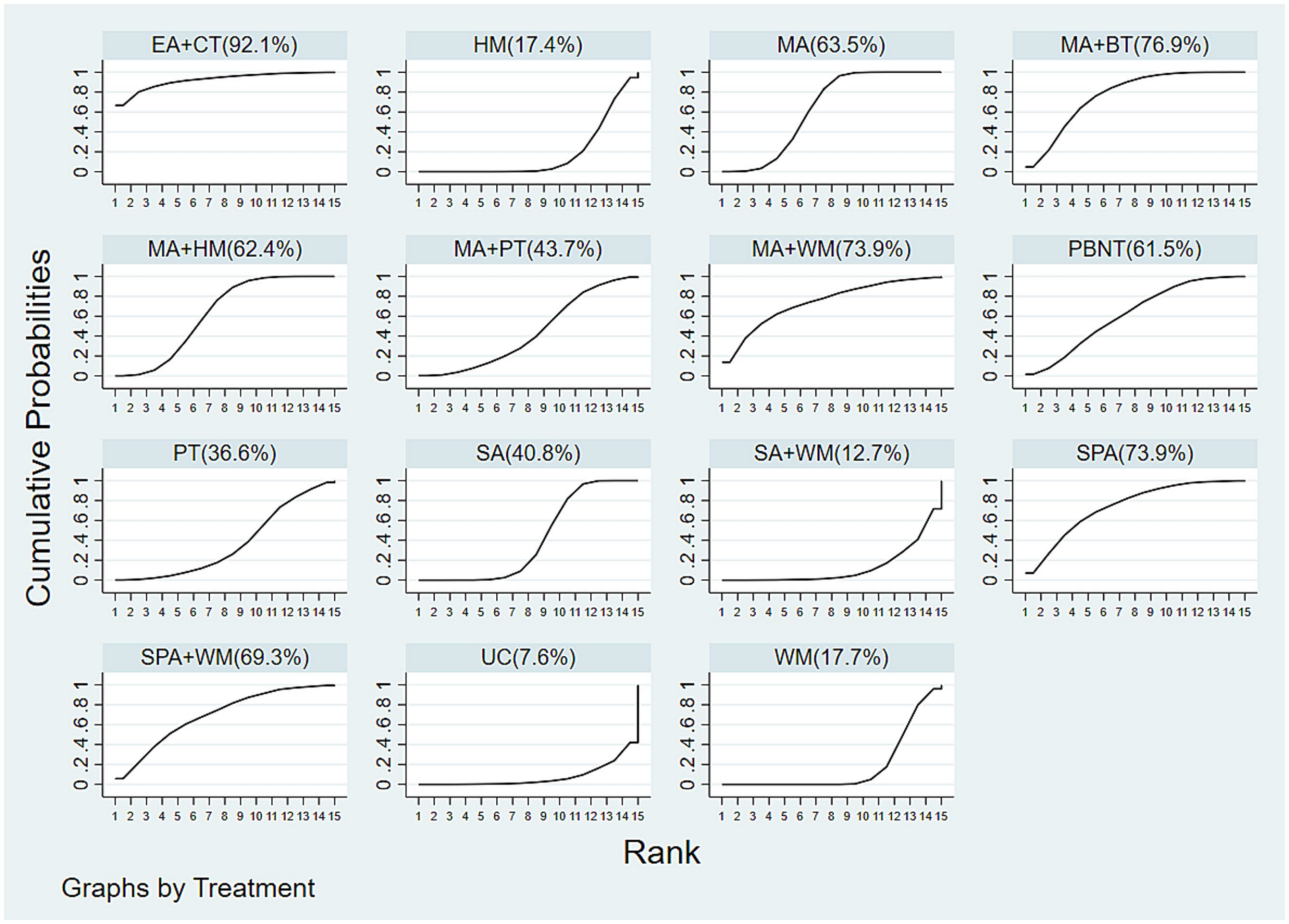
Cumulative probability ranking results of responder rate.

The cumulative probability rankings for responder rate are as follows: EA + CT (92.1%) > MA + BT (76.9%) > MA + WM (73.9%) = SPA (73.9%) > SPA + WM (69.3%) > MA (63.5%) > MA + HM (62.4%) > PBNT (61.5%) > MA + PT (43.7%) > SA (40.8%) > PT (36.6%) > WM (17.7%) > HM (17.4%) > SA + WM (12.7%) > UC (7.6%), as shown in Figure 9.

Funnel plot analysis conducted using Stata 17.0 is included in the Supplementary Figure S4. The funnel plot exhibits some asymmetry, which suggests the presence of potential publication bias or small-study effects within the research network.

#### 3.4.5 Adverse reaction

A total of 31 studies evaluated adverse reactions, among which 27 reported no adverse events, while the remaining 4 studies documented related adverse reactions. The most commonly reported acupuncture-related adverse effects included subcutaneous hematoma, pain around the needling site, headache exacerbation, temporary headache triggered by needling, and acupuncture-induced syncope.

## 4 Discussion

The etiology and pathogenesis of chronic TTH remain incompletely understood. Potential contributing factors include persistent muscle tension in the head and neck, leading to local ischemia and the accumulation of metabolic byproducts, which may trigger pain. Other factors involve peripheral and central sensitization, resulting in heightened sensitivity to pain and stress, thus perpetuating headaches. Imbalances in neurotransmitters can lower pain thresholds and amplify pain perception. Psychosocial factors, including negative emotional states and lifestyle habits, may exacerbate headaches indirectly by increasing muscle tension and affecting neurotransmitter levels (1, 24).

In this study, we conducted an extensive literature review and included 42 RCTs that met our inclusion criteria, covering 21 different treatment methods—13 single therapies and 8 combination therapies. Our evaluation of various acupuncture techniques focused on headache frequency, duration, pain intensity, and responder rate, leading to the following conclusions:

1. Responder rate: As a primary outcome, responder rate provides a direct measure of treatment efficacy. Our analysis showed that EA + CT demonstrating the highest efficacy.
2. Headache frequency: EA was found to be the most effective in reducing headache frequency. In addition, combination therapies generally exceeded the efficacy of single treatments with MA, PBNT, or WM.
3. Headache duration: EA emerged as potentially the most effective treatment for shortening headache duration.
4. Pain intensity: The MA + WM combination was superior to other therapies in reducing pain intensity. Combinations of MA with WM or HM were more effective than MA, WM, or HM alone.
5. Adverse reactions: Among the included studies, Endres et al. (25) reported one case of severe headache potentially triggered by sham acupuncture, while no further evidence was provided. Regarding the other three studies reported adverse events, the typical ones include localized pain, bruising, and acupuncture syncope, which usually resolve quickly without further intervention. Therefore, the relatively low incidence and mild effects of the adverse reactions reported in included studies reveals that the safety profile of acupuncture is generally favorable.

EA frequently appeared in our results, particularly as it achieved the best outcomes in responder rate. The mechanism behind EA combined with CT may involve its synergy with cupping therapy. EA applies mild electrical currents to specific acupoints, enhancing local blood circulation and regulating the nervous system. Research indicates that EA increases endogenous opioid release, inhibits pain signal transmission, and alleviates headaches (15). Additionally, EA can balance sympathetic and parasympathetic nervous systems, reducing muscle tension and anxiety, which in turn improves headache symptoms (26). Cupping therapy, through negative pressure, promotes blood and lymphatic circulation, reducing local muscle tension and inflammatory responses. Its mechanical stimulation enhances tissue oxygenation and metabolism and facilitates the removal of harmful substances. Furthermore, cupping therapy stimulates skin and muscle nerve endings, modulating central nervous system functions and alleviating pain and discomfort (27).

EA integrates electrical stimulation with traditional acupuncture needling, providing a more standardized and reproducible approach compared to manual manipulation. Key EA parameters include waveform, frequency, and intensity, with waveform being one of the most critical factors. Commonly used waveforms are the dense-sparse (alternating dense and sparse pulses) and continuous waveforms. The dense-sparse waveform alternates between rapid and slower pulses, effectively promoting rhythmic muscle contraction and relaxation, which enhances circulation, reduces edema, and provides analgesic effects.

Although clinical studies specifically examining the impact of EA waveforms on TTH are limited, existing literature suggests the dense-sparse waveform demonstrates promising results in pain and edema reduction across various conditions. For instance, Wu et al. (28) explored the efficacy of different EA waveforms in treating primary dysmenorrhea and found no significant difference in short- and long-term pain relief or reduction in analgesic use across waveforms, although the dense-sparse waveform showed a slight advantage in immediate pain relief. In a RCT of EA waveform effects on 60 patients with cervical spondylosis, Hu et al. (29) reported that the dense-sparse waveform significantly alleviated dizziness and headaches compared to the continuous wave, highlighting its potential benefits.

These findings suggest that the dense-sparse waveform may facilitate rhythmic contraction and relaxation of cranio-cervical muscles, thereby improving blood and lymphatic circulation and potentially relieving TTH. However, further studies are necessary to verify this hypothesis and establish optimal parameters for clinical efficacy.

When comparing our findings with those of Hu et al. (30), we noted discrepancies. Hu et al. identified SPA + WM as most effective for reducing pain intensity, whereas our study found MA + WM to be optimal. They reported MA + HM as effective in reducing headache frequency, while EA was found to be superior in our analysis. For responder rate, Hu et al. favored BT, while EA + CT was most effective in our study. Possible reasons for these differences include sample size variations—Hu et al. included 27 studies primarily from China and Asia, while our study incorporated 42 studies featuring a diverse range of research teams from nine different countries, including China, Germany, Sweden, and others. The majority of the populations studied were from Asia, while the research also encompassed data from Europe, North America, and the Middle East. Additionally, there is a broader range of treatments in our study, including LA, RT, and PT, which were not covered by Hu et al.

Through comprehensive search of 8 databases, this study represents the latest network meta-analysis evaluating various acupuncture methods for TTH, incorporating the highest number of studies, the most recent data, and a wide array of treatment modalities.

Despite the valuable insights provided, this study has limitations. Firstly, some included studies did not report on allocation concealment, blinding, or selective reporting biases, which may introduce bias. Secondly, smaller sample sizes in some studies might affect result accuracy. Lastly, although the study involved global research teams, a substantial proportion of participants were Asian, which may impact the generalizability of the findings to other ethnic groups.

In conclusion, our study demonstrates that among the 21 treatments assessed, EA is most effective in reducing headache frequency and duration, while EA + CT and MA + WM are the most effective for improving responder rate and reducing pain intensity, respectively. However, clinical decisions should consider individual patient circumstances. Given the current limitations in literature, further multi-center, large-scale, and prospective RCTs are necessary to confirm these findings.

## Data Availability

The original contributions presented in the study are included in the article/Supplementary material, further inquiries can be directed to the corresponding author.

## Footnotes

^1^https://www.crd.york.ac.uk/prospero/#recordDetails
