# Supplementary material for: Efficacy of different acupuncture-related therapies for tension-type headache: a systematic review and network meta-analysis
Source: Front Neurol. 2024 Dec 5;15:1481715. doi: 10.3389/fneur.2024.1481715 (PMC11655348; doi:10.3389/fneur.2024.1481715)
Supplement: Supplementary file 1 [file Table_1.docx]

Table S1. PRISMA NMA Checklist of Items to Include When Reporting a Systematic Review Involving a Network Meta-analysis

| **Section/Topic** | **Item #** | **Checklist Item** | **Reported in Section #** |
| --- | --- | --- | --- |
| **TITLE** |  |  |  |
| Title | 1 | Identify the report as a systematic review *incorporating*  *anetwork meta-analysis (or related form of meta-analysis).* | **Title** |
|  |  |  |  |
| **ABSTRACT** |  |  |  |
| Structured summary | 2 | Provide a structured summary including, as applicable:  **Background:** main objectives  **Methods:** data sources; study eligibility criteria, participants, and interventions; study appraisal; and *synthesis methods, such as network meta-analysis.*  **Results:** number of studies and participants identified; summary estimates with corresponding confidence/credible intervals; *treatment rankings may also be discussed. Authors may choose to summarize pairwise comparisons against a chosen treatment included in their analyses for brevity.*  **Discussion/Conclusions:** limitations; conclusions and implications of findings.  **Other:** systematic review registration number with registry name. | Abstract |
|  |  |  |  |
| **INTRODUCTION** |  |  |  |
| Rationale | 3 | Describe the rationale for the review in the context of what is already known*, including mention of why a network meta-analysis has been conducted.* | Abstract |
| Objectives | 4 | Provide an explicit statement of questions being addressed, with reference to participants, interventions, comparisons, outcomes, and study design (PICOS). | Abstract |
|  |  |  |  |
| **METHODS** |  |  |  |
| Protocol and registration | 5 | Indicate whether a review protocol exists and if and where it can be accessed (e.g., Web address); and, if available, provide registration information, including registration number. | **2.1** |
| Eligibility criteria | 6 | Specify study characteristics (e.g., PICOS, length of follow-up) and report characteristics (e.g., years considered, language, publication status) used as criteria for eligibility, giving rationale. *Clearly describe eligible treatments included in the treatment network, and note whether any have been clustered or merged into the same node (with justification).* | **2.3.1** |
| Information sources | 7 | Describe all information sources (e.g., databases with dates of coverage, contact with study authors to identify additional studies) in the search and date last searched. | **2.2** |
| Search | 8 | Present full electronic search strategy for at least one database, including any limits used, such that it could be repeated. | 2.2  Supplementary  TableS2 |
| Study selection | 9 | State the process for selecting studies (i.e., screening, eligibility, included in systematic review, and, if applicable, included in the meta-analysis). | 2.3.1；2.3.2 |
| Data collection process | 10 | Describe method of data extraction from reports (e.g., piloted forms, independently, in duplicate) and any processes for obtaining and confirming data from investigators. | 2.5 |
| Data items | 11 | List and define all variables for which data were sought (e.g., PICOS, funding sources) and any assumptions and simplifications made. | **2.5** |
| **Geometry of the network** | **S1** | Describe methods used to explore the geometry of the treatment network under study and potential biases related to it. This should include how the evidence base has been graphically summarized for presentation, and what characteristics were compiled and used to describe the evidence base to readers. | 2.7 |
| Risk of bias within individual studies | 12 | Describe methods used for assessing risk of bias of individual studies (including specification of whether this was done at the study or outcome level), and how this information is to be used in any data synthesis. | 2.6 |
| Summary measures | 13 | State the principal summary measures (e.g., risk ratio, difference in means). *Also describe the use of additional summary measures assessed, such as treatment rankings and surface under the cumulative ranking curve (SUCRA) values, as well as modified approaches used to present summary findings from meta-analyses.* | 2.7 |
| Planned methods of analysis | 14 | Describe the methods of handling data and combining results of studies for each network meta-analysis. This should include, but not be limited to:   - *Handling of multi-arm trials;* - *Selection of variance structure;* - *Selection of prior distributions in Bayesian analyses; and* - *Assessment of model fit.* | 2.7 |
| **Assessment of Inconsistency** | **S2** | Describe the statistical methods used to evaluate the agreement of direct and indirect evidence in the treatment network(s) studied. Describe efforts taken to address its presence when found. | 2.7 |
| Risk of bias across studies | 15 | Specify any assessment of risk of bias that may affect the cumulative evidence (e.g., publication bias, selective reporting within studies). | **2.6** |
| Additional analyses | 16 | Describe methods of additional analyses if done, indicating which were pre-specified. This may include, but not be limited to, the following:   - Sensitivity or subgroup analyses; - Meta-regression analyses; - *Alternative formulations of the treatment network; and* - *Use of alternative prior distributions for Bayesian analyses (if applicable).* | **2.7** |
|  |  |  |  |
| **RESULTS** |  |  |  |
| Study selection | 17 | Give numbers of studies screened, assessed for eligibility, and included in the review, with reasons for exclusions at each stage, ideally with a flow diagram. | **3.1** |
| **Presentation of network structure** | **S3** | Provide a network graph of the included studies to enable visualization of the geometry of the treatment network. | **Figure3** |
| **Summary of network geometry** | **S4** | Provide a brief overview of characteristics of the treatment network. This may include commentary on the abundance of trials and randomized patients for the different interventions and pairwise comparisons in the network, gaps of evidence in the treatment network, and potential biases reflected by the network structure. | **3.2** |
| Study characteristics | 18 | For each study, present characteristics for which data were extracted (e.g., study size, PICOS, follow-up period) and provide the citations. | 3.2 |
| Risk of bias within studies | 19 | Present data on risk of bias of each study and, if available, any outcome level assessment. | **3.3** |
| Results of individual studies | 20 | For all outcomes considered (benefits or harms), present, for each study: 1) simple summary data for each intervention group, and 2) effect estimates and confidence intervals. *Modified approaches may be needed to deal with information from larger networks.* | 3.4 |
| Synthesis of results | 21 | Present results of each meta-analysis done, including confidence/credible intervals. *In larger networks, authors may focus on comparisons versus a particular comparator (e.g. placebo or standard care), with full findings presented in an appendix. League tables and forest plots may be considered to summarize pairwise comparisons.* If additional summary measures were explored (such as treatment rankings), these should also be presented. | **3.4** |
| **Exploration for inconsistency** | **S5** | Describe results from investigations of inconsistency. This may include such information as measures of model fit to compare consistency and inconsistency models, *P* values from statistical tests, or summary of inconsistency estimates from different parts of the treatment network. | 3.4 |
| Risk of bias across studies | 22 | Present results of any assessment of risk of bias across studies for the evidence base being studied. | 3.3 |
| Results of additional analyses | 23 | Give results of additional analyses, if done (e.g., sensitivity or subgroup analyses, meta-regression analyses, alternative network geometries studied, alternative choice of prior distributions for Bayesian analyses, and so forth). | **3.4** |
|  |  |  |  |
| **DISCUSSION** |  |  |  |
| Summary of evidence | 24 | Summarize the main findings, including the strength of evidence for each main outcome; consider their relevance to key groups (e.g., healthcare providers, users, and policy-makers). | **4** |
| Limitations | 25 | Discuss limitations at study and outcome level (e.g., risk of bias), and at review level (e.g., incomplete retrieval of identified research, reporting bias). *Comment on the validity of the assumptions, such as transitivity and consistency. Comment on any concerns regarding network geometry (e.g., avoidance of certain comparisons).* | 4 |
| Conclusions | 26 | Provide a general interpretation of the results in the context of other evidence, and implications for future research. | **4** |
|  |  |  |  |
| **FUNDING** |  |  |  |
| Funding | 27 | Describe sources of funding for the systematic review and other support (e.g., supply of data); role of funders for the systematic review. This should also include information regarding whether funding has been received from manufacturers of treatments in the network and/or whether some of the authors are content experts with professional conflicts of interest that could affect use of treatments in the network. | ***Funding*** |

PICOS = population, intervention, comparators, outcomes, study design.

* Text in italics indicate S wording specific to reporting of network meta-analyses that has been added to guidance from the PRISMA statement.

† Authors may wish to plan for use of appendices to present all relevant information in full detail for items in this section.

Table S2. Literature Search Strategy

Search strategy for English database

Search Keywords:

1: acupuncture OR electroacupuncture OR warm needle acupuncture OR milli needles OR filiform needle OR acupoint injection OR acupoint catgut embedding

OR catgut implantation OR scalp acupuncture OR scalp needle OR manual acupuncture OR ear acupuncture OR auricular acupuncture OR ear needle OR auriculotherapy OR acupoints OR acupuncture points OR acupressure OR electric stimulation therapy OR electric pulse stimulation OR laser acupuncture

2: tension-type headache OR TTH OR idiopathic headache OR stress headache OR psychogenic headache OR tension-vascular headache OR tension headache OR headache OR headache disorder OR head pain OR cephalalgia OR cephalodynia OR cranial pain OR cerebral pain

3: randomized controlled trial OR randomized OR placebo

**Table S1 The detailed search strategy for all four databases incl. search terms used for the systematic literature searches**

| **Database** | **Search Term** | **Result** |
| --- | --- | --- |
| **PubMed** | #1: "Acupuncture"[Title/Abstract] OR "electroacupuncture"[Title/Abstract] OR "warm needle acupuncture"[Title/Abstract] OR ("milli"[All Fields] AND "needles"[Title/Abstract]) OR "filiform needle"[Title/Abstract] OR "acupoint injection"[Title/Abstract] OR "acupoint catgut embedding"[Title/Abstract] OR "catgut implantation"[Title/Abstract] OR "scalp acupuncture"[Title/Abstract] OR "scalp needle"[Title/Abstract] OR "manual acupuncture"[Title/Abstract] OR "ear acupuncture"[Title/Abstract] OR "auricular acupuncture"[Title/Abstract] OR (("ear"[MeSH Terms] OR "ear"[All Fields]) AND "needle"[Title/Abstract]) OR "auriculotherapy"[Title/Abstract] OR "acupoints"[Title/Abstract] OR "acupuncture points"[Title/Abstract] OR "acupressure"[Title/Abstract] OR "electric stimulation therapy"[Title/Abstract] OR "electric pulse stimulation"[Title/Abstract] OR "laser acupuncture"[Title/Abstract] OR "Acupuncture"[MeSH Terms] | 36029 |
|  | #2: "Tension-Type Headache"[Title/Abstract] OR "TTH"[Title/Abstract] OR "idiopathic headache"[Title/Abstract] OR "stress headache"[Title/Abstract] OR "psychogenic headache"[Title/Abstract] OR "tension vascular headache"[Title/Abstract] OR "tension headache"[Title/Abstract] OR "headache"[Title/Abstract] OR "headache disorder"[Title/Abstract] OR "head pain"[Title/Abstract] OR "cephalalgia"[Title/Abstract] OR "cephalodynia"[Title/Abstract] OR "cranial pain"[Title/Abstract] OR "cerebral pain"[Title/Abstract] OR "Tension-Type Headache"[MeSH Terms] | 93530 |
|  | #3: randomized controlled trial"[Publication Type] OR "randomized"[Title/Abstract] OR "placebo"[Title/Abstract] | 1057652 |
|  | #4: #1 AND #2 AND #3 | **301** |
| **Web of Science**  **( Title )** | #1: TI=(acupuncture OR electroacupuncture OR warm needle acupuncture OR milli needles OR filiform needle OR acupoint injection OR acupoint catgut embedding OR catgut implantation OR scalp acupuncture OR scalp needle OR manual acupuncture OR ear acupuncture OR auricular acupuncture OR ear needle OR auriculotherapy OR acupoints OR acupuncture points OR acupressure OR electric stimulation therapy OR electric pulse stimulation OR laser acupuncture) | 18179 |
|  | #2: TI=(tension-type headache OR TTH OR idiopathic headache OR stress headache OR psychogenic headache OR tension-vascular headache OR tension headache OR headache OR headache disorder OR head pain OR cephalalgia OR cephalodynia OR cranial pain OR cerebral pain) | 29071 |
|  | #3: TI=(randomized controlled trial OR randomized OR placebo) | 378541 |
|  | #4: #1 AND #2 AND #3 | 48 |
| **Web of Science**  **( Abstract )** | #5: AB=(acupuncture OR electroacupuncture OR warm needle acupuncture OR milli needles OR filiform needle OR acupoint injection OR acupoint catgut embedding OR catgut implantation OR scalp acupuncture OR scalp needle OR manual acupuncture OR ear acupuncture OR auricular acupuncture OR ear needle OR auriculotherapy OR acupoints OR acupuncture points OR acupressure OR electric stimulation therapy OR electric pulse stimulation OR laser acupuncture) | 22710 |
|  | #6: AB=(tension-type headache OR TTH OR idiopathic headache OR stress headache OR psychogenic headache OR tension-vascular headache OR tension headache OR headache OR headache disorder OR head pain OR cephalalgia OR cephalodynia OR cranial pain OR cerebral pain) | 94226 |
|  | #7: AB=( randomized controlled trial OR randomized OR placebo) | 831008 |
|  | #8: #5 AND #6 AND #7 | 280 |
|  | #9: #4 OR #8 | **290** |
| **EMBASE** | #1: acupuncture:ab,ti OR electroacupuncture:ab,ti OR 'warm needle acupuncture':ab,ti OR 'milli needles':ab,ti OR 'filiform needle':ab,ti OR 'acupoint injection':ab,ti OR 'acupoint catgut embedding':ab,ti OR 'catgut implantation':ab,ti OR 'scalp acupuncture':ab,ti OR 'scalp needle':ab,ti OR 'manual acupuncture':ab,ti OR 'ear acupuncture':ab,ti OR 'auricular acupuncture':ab,ti OR 'ear needle':ab,ti OR auriculotherapy:ab,ti OR acupoints:ab,ti OR 'acupuncture points':ab,ti OR acupressure:ab,ti OR 'electric stimulation therapy':ab,ti OR 'electric pulse stimulation':ab,ti OR 'laser acupuncture':ab,ti | 47277 |
|  | #1’: 'acupuncture'/exp OR 'acupuncture' | 71463 |
|  | #1’’: #1 OR #1’ | 71980 |
|  | #2: 'tension-type headache':ab,ti OR tth:ab,ti OR 'idiopathic headache':ab,ti OR 'stress headache':ab,ti OR 'psychogenic headache':ab,ti OR 'tension-vascular headache':ab,ti OR 'tension headache':ab,ti OR headache:ab,ti OR 'headache disorder':ab,ti OR 'head pain':ab,ti OR cephalalgia:ab,ti OR cephalodynia:ab,ti OR 'cranial pain':ab,ti OR 'cerebral pain':ab,ti | 150531 |
|  | #2’: 'tension headache'/exp OR 'tension headache' | 9754 |
|  | #2’’: #2 OR #2’ | 152256 |
|  | #3: 'randomized controlled trial'/exp OR 'randomized controlled trial' | 1108356 |
|  | #4: #1’’ AND #2’’ AND #3 | **395** |
| **Cochrane Library** | #1: (acupuncture OR electroacupuncture OR (warm needle acupuncture) OR (milli needles) OR (filiform needle) OR (acupoint injection) OR (acupoint catgut embedding) OR (catgut implantation) OR (scalp acupuncture) OR (scalp needle) OR (manual acupuncture) OR (ear acupuncture) OR (auricular acupuncture) OR (ear needle) OR auriculotherapy OR acupoints OR (acupuncture points) OR acupressure OR (electric stimulation therapy) OR (electric pulse stimulation) OR (laser acupuncture)):ti,ab,kw | 33167 |
|  | #1’: MeSH descriptor: [Acupuncture] explode all trees | 224 |
|  | #1’’: #1 OR #1’ | 33167 |
|  | #2: ((tension-type headache) OR TTH OR (idiopathic headache) OR (stress headache) OR (psychogenic headache) OR (tension-vascular headache) OR (tension headache) OR headache OR (headache disorder) OR (head pain) OR cephalalgia OR cephalodynia OR (cranial pain) OR (cerebral pain)):ti,ab,kw | 45650 |
|  | #2’: MeSH descriptor: [Tension-Type Headache] explode all trees | 417 |
|  | #2’’: #2 OR #2’ | 45650 |
|  | #3: #1’’ AND #2’’ | **1456** |
| **In all** | **2442** | |
| **Time** | **19 April 2024** | |

Search strategy for Chinese database

Search Keywords:

1: 针刺 OR 针灸 OR 电针 OR 头针 OR 耳针 OR 温针灸 OR 穴位埋针 OR 穴位按压 OR 电脉冲刺激 OR 镭射针灸

2: 紧张型头痛 OR 压力性头痛 OR 血管性头痛 OR 特发性头痛 OR 头痛

| **Database** | **Search Term** | **Result** |
| --- | --- | --- |
| **Wanfang** | 主题:(紧张型头痛 OR 压力性头痛 OR 血管性头痛 OR 特发性头痛 OR 头痛) AND 主题:(针刺 OR 针灸 OR 电针 OR 头针 OR 耳针 OR 温针灸 OR 穴位埋针 OR 穴位按压 OR 电脉冲刺激 OR 镭射针灸) | **373** |
| **VIP** | [((((((((((题名或关键词=针刺 OR 题名或关键词=针灸) OR 题名或关键词=电针) OR 题名或关键词=头针) OR 题名或关键词=耳针) OR 题名或关键词=温针灸) OR 题名或关键词=穴位埋针) OR 题名或关键词=穴位按压) OR 题名或关键词=电脉冲刺激) OR 题名或关键词=镭射针灸) AND ((((题名或关键词=紧张型头痛 OR 题名或关键词=压力性头痛) OR 题名或关键词=血管性头痛) OR 题名或关键词=特发性头痛) OR 题名或关键词=头痛))](https://qikan.cqvip.com/Qikan/search/index?LngMySearHistoryIdGuid=cc2dbf36-53a0-49ac-90a3-35ac907073be&from=Qikan_Article_History) | **3160** |
| **CNKI** | ( ( ( ( ( ( ( ( ( ( ( ( 主题%='针刺' or 主题%='針刺' or 题名%='针刺' or 题名%='針刺' ) OR ( 主题%='针灸' or 主题%='針灸' or 题名%='针灸' or 题名%='針灸' ) ) OR ( 主题%='电针' or 主题%='電針' or 题名%='电针' or 题名%='電針' ) ) OR ( 主题%='头针' or 主题%='頭針' or 题名%='头针' or 题名%='頭針' ) ) OR ( 主题%='耳针' or 主题%='耳針' or 题名%='耳针' or 题名%='耳針' ) ) OR ( 主题%='温针灸' or 主题%='溫針灸' or 题名%='温针灸' or 题名%='溫針灸' ) ) OR ( 主题%='穴位埋针' or 主题%='穴位埋針' or 题名%='穴位埋针' or 题名%='穴位埋針' ) ) OR ( 主题%='穴位按压' or 主题%='穴位按壓' or 题名%='穴位按压' or 题名%='穴位按壓' ) ) OR ( 主题%='电脉冲刺激' or 主题%='電脈沖刺激' or 题名%='电脉冲刺激' or 题名%='電脈沖刺激' ) ) OR ( 主题%='镭射针灸' or 主题%='鐳射針灸' or 题名%='镭射针灸' or 题名%='鐳射針灸' ) ) AND ( ( ( ( ( ( ( ( ( ( 主题%='针刺' or 主题%='針刺' or 题名%='针刺' or 题名%='針刺' ) OR ( 主题%='针灸' or 主题%='針灸' or 题名%='针灸' or 题名%='針灸' ) ) OR ( 主题%='电针' or 主题%='電針' or 题名%='电针' or 题名%='電針' ) ) OR ( 主题%='头针' or 主题%='頭針' or 题名%='头针' or 题名%='頭針' ) ) OR ( 主题%='耳针' or 主题%='耳針' or 题名%='耳针' or 题名%='耳針' ) ) OR ( 主题%='温针灸' or 主题%='溫針灸' or 题名%='温针灸' or 题名%='溫針灸' ) ) OR ( 主题%='穴位埋针' or 主题%='穴位埋針' or 题名%='穴位埋针' or 题名%='穴位埋針' ) ) OR ( 主题%='穴位按压' or 主题%='穴位按壓' or 题名%='穴位按压' or 题名%='穴位按壓' ) ) OR ( 主题%='电脉冲刺激' or 主题%='電脈沖刺激' or 题名%='电脉冲刺激' or 题名%='電脈沖刺激' ) ) OR ( 主题%='镭射针灸' or 主题%='鐳射針灸' or 题名%='镭射针灸' or 题名%='鐳射針灸' ) ) ) AND ( ( ( ( ( 主题%='紧张型头痛' or 主题%='緊張型頭痛' or 题名%='紧张型头痛' or 题名%='緊張型頭痛' ) OR ( 主题%='压力性头痛' or 主题%='壓力性頭痛' or 题名%='压力性头痛' or 题名%='壓力性頭痛' ) ) OR ( 主题%='血管性头痛' or 主题%='血管性頭痛' or 题名%='血管性头痛' or 题名%='血管性頭痛' ) ) OR ( 主题%='特发性头痛' or 主题%='特發性頭痛' or 题名%='特发性头痛' or 题名%='特發性頭痛' ) ) OR ( 主题%='头痛' or 主题%='頭痛' or 题名%='头痛' or 题名%='頭痛' ) ) )  [(紧张型头痛 OR 压力性头痛 OR 血管性头痛 OR 特发性头痛 OR 头痛) AND (针刺 OR 针灸 OR 电针 OR 头针 OR 耳针 OR 温针灸 OR 穴位埋针 OR 穴位按压 OR 电脉冲刺激 OR 镭射针灸)](javascript:toDoRelimitSearch();) | **2953** |
| **CBM** | [( "针刺"[标题] OR "针灸"[标题] OR "电针"[标题] OR "头针"[标题] OR "耳针"[标题] OR "温针灸"[标题] OR "穴位埋针"[标题] OR "穴位按压"[标题] OR "电脉冲刺激"[标题] OR "镭射针灸"[标题]) AND( "紧张型头痛"[标题] OR "压力性头痛"[标题] OR "血管性头痛"[标题] OR "特发性头痛"[标题] OR "头痛"[标题])](javascript:toDoRelimitSearch();) | **2238** |
| **In all** | **8724** |  |
| **Time** | **19 April 2024** |  |

Table S3. Excluded studies

| **No.** | **First Author and**  **Year of**  **publication** | **Title** | **Excluded Reason** |
| --- | --- | --- | --- |
| 1 | Serhat Koran.2021 | Search for the clinical effectiveness of Korean Tae-Geuk acupuncture therapy in chronic tension-type headache | Unavailable data |
| 2 | Sıla Gildir.2019 | A randomized trial of trigger point dry needling versus sham needling for chronic tension-type headache | Acupuncture was not the main intervention |
| 3 | Wildete Carvalho Mayrink.2018 | Effectiveness of Acupuncture as Auxiliary Treatment for Chronic Headache | TTH was not the main research subject |
| 4 | ALBRECHT F. MOLSBERGER.2006 | Designing an Acupuncture Study: The Nationwide, Randomized, Controlled, German Acupuncture Trials on Migraine and Tension-Type Headache | A protocol |
| 5 | AJ Vickers.2004 | Acupuncture of chronic headache disorders in primary care: randomised controlled trial and economic analysis | TTH was not the main research subject |
| 6 | David Wonderling.2004 | Cost effectiveness analysis of a randomised trial of acupuncture for chronic headache in primary care | TTH was not the main research subject |
| 7 | Andrew J Vickers.2004 | Acupuncture for chronic headache in primary care: large, pragmatic, randomised trial | TTH was not the main research subject |
| 8 | M. Fink.2001 | Credibility of a Newly Designed Placebo Needle for Clinical Trials in Acupuncture Research | Research on credibility of placebo |
| 9 | Kevanr.Wylie.1997 | Does psychological testing help to predict the response to acupuncture or massage/relaxation therapy in patients presenting to a general neurology clinic with headache | Mainly focused on the effectiveness of psychological tests |
| 10 | AR White.1996 | A Pilot Study of Acupuncture for Tension Headache, Using a Novel Placebo | A pilot study |
| 11 | C. A. VINCENT.1990 | THE TREATMENT OF TENSION HEADACHE BY ACUPUNCTURE: A CONTROLLED SINGLE CASE DESIGN WITH TIME SERIES ANALYSIS | A single case design |
| 12 | Denise Millstine.2017 | Complementary and integrative medicine in the management of headache | A review |
| 13 | George Georgoudis.2017 | The effect of myofascial release and microwave diathermy combined with acupuncture versus acupuncture therapy in tension‐type headache patients: A pragmatic randomized controlled trial | Acupuncture was not the main intervention |
| 14 | S Jena.2008 | Acupuncture in patients with headache | TTH was not the main research subject |
| 15 | Remy R. Coeytaux.2005 | A Randomized, Controlled Trial of Acupuncture for Chronic Daily Headache | TTH was not the main research subject |
| 16 | L LOH.1984 | Acupuncture versus medical treatment for migraine and muscle tension headaches | TTH was not the main research subject |
| 17 | Esa Ahonen.1984 | EFFECTIVENESS OF ACUPUNCTURE AND PHYSIOTHERAPY ON MYOGENIC HEADACHE: A COMPARATIVE STUDY | TTH was not the main research subject |
| 18 | Joerg Schiller.2022 | Effects of acupuncture and medical training therapy on depression, anxiety, and quality of life in patients with frequent tension-typeheadache: A randomized controlled study | Unavailable data |
| 19 | Elisabeth I. So ̈derberg.2011 | Subjective Well-being in Patients With Chronic Tension-type Headache: Effect of Acupuncture, Physical Training and Relaxation Training | Unavailable data |
| 20 | Jane Carlsson.1990 | Health Status in Patients with Tension Headache Treated with Acupuncture or Physiotherapy | Unavailable data |
| 21 | Tiziana Tavola.1991 | Traditional Chinese acupuncture in tension-type headache: a controlled study | Unavailable data |
| 22 | Jane Carlsson.1990 | Muscle tenderness in tension headache treated with acupuncture or physiotherapy | Unavailable data |
| 23 | Chen.2010 | Clinical study on the treatment of tension-type headache by plum blossom needle tapping against needle acupuncture | Unavailable data |
| 24 | Xu.2000 | Observation of the therapeutic effects of acupuncture compared with Western medicine in the treatment of cervicogenic headache | TTH was not the main research subject |
| 25 | Li.2004 | Wrist-ankle acupuncture for tension headache 30 cases | Different diagnostic criteria |
| 26 | Zhang.2011 | Effect of Tiaoshen Shugan acupuncture on tension-type headache and its influence on serum adiponectin content | Unavailable data |


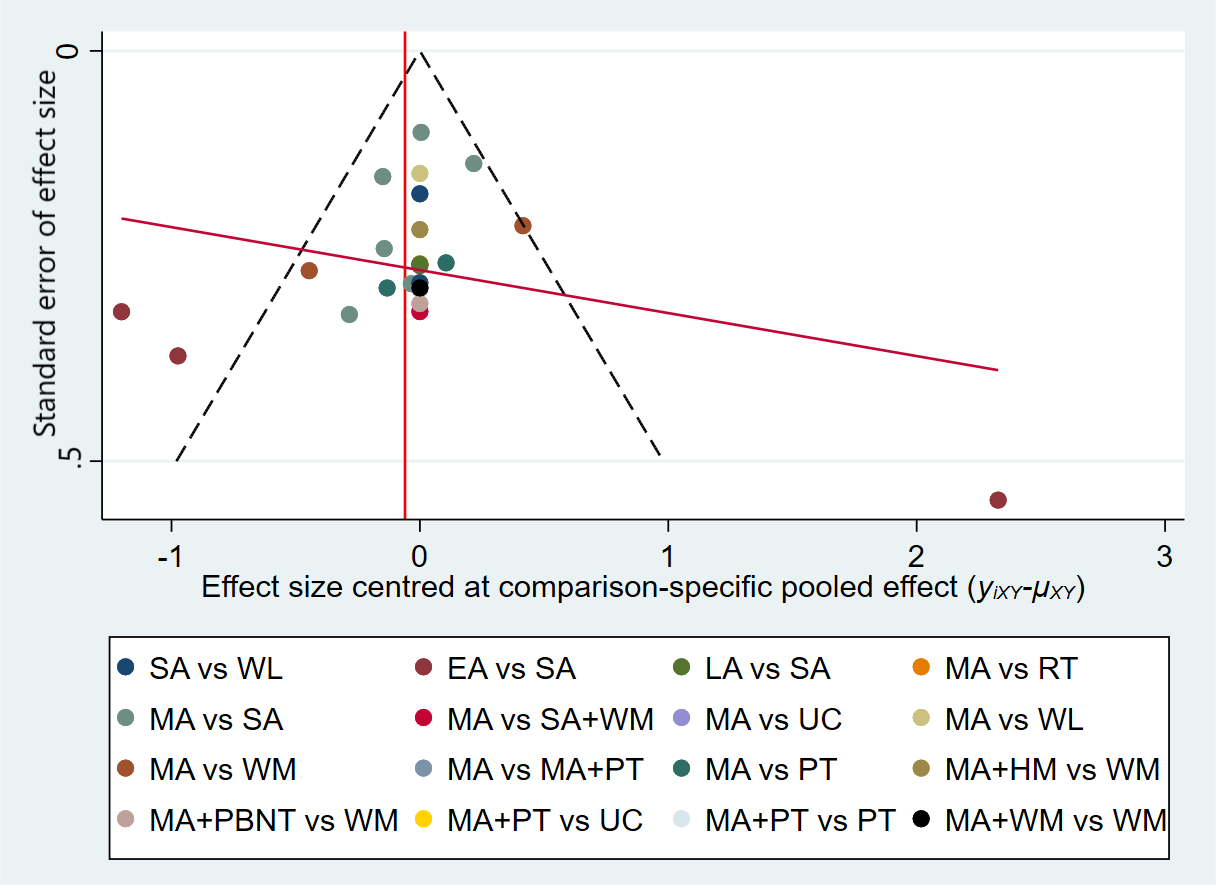


**Figure S1. Funnel plot of headache frequency**

**
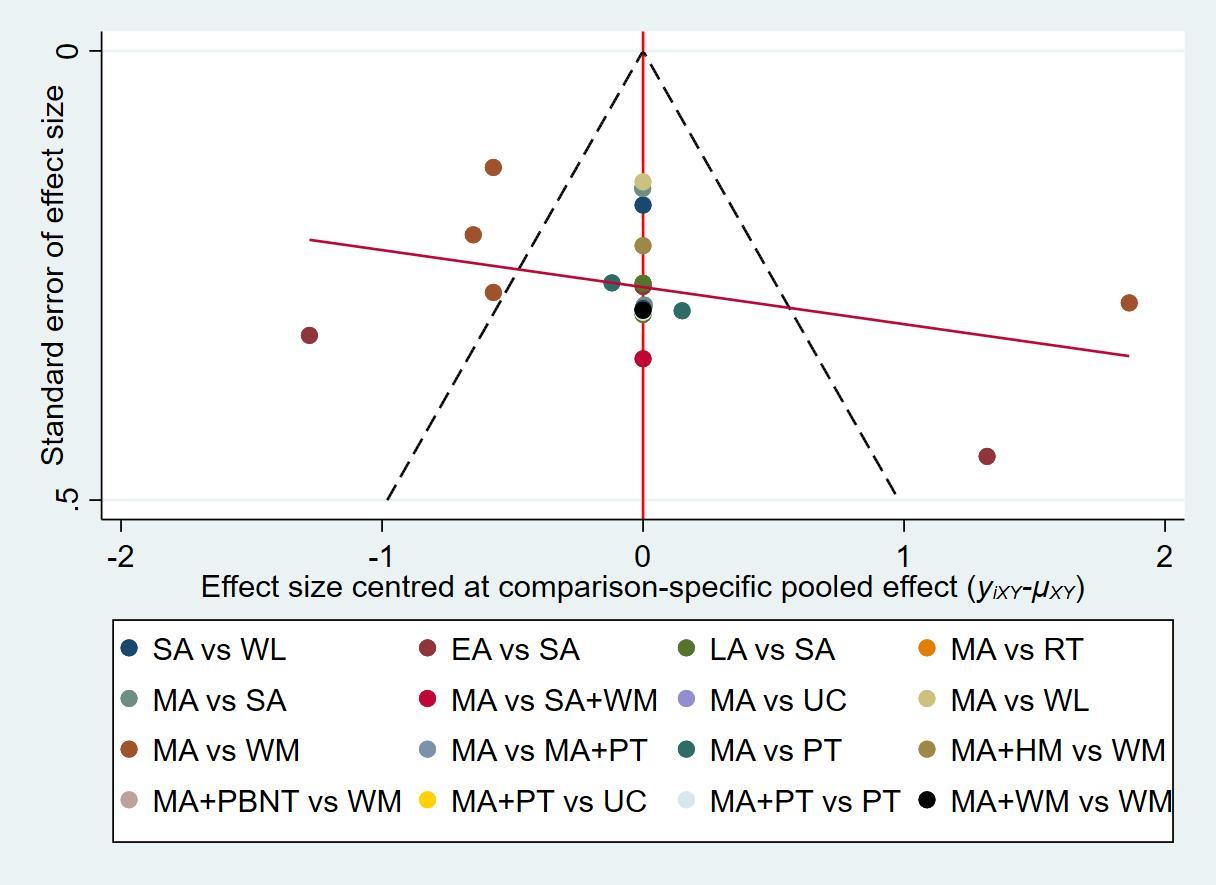
**

**Figure S2. Funnel plot of headache duration**

**
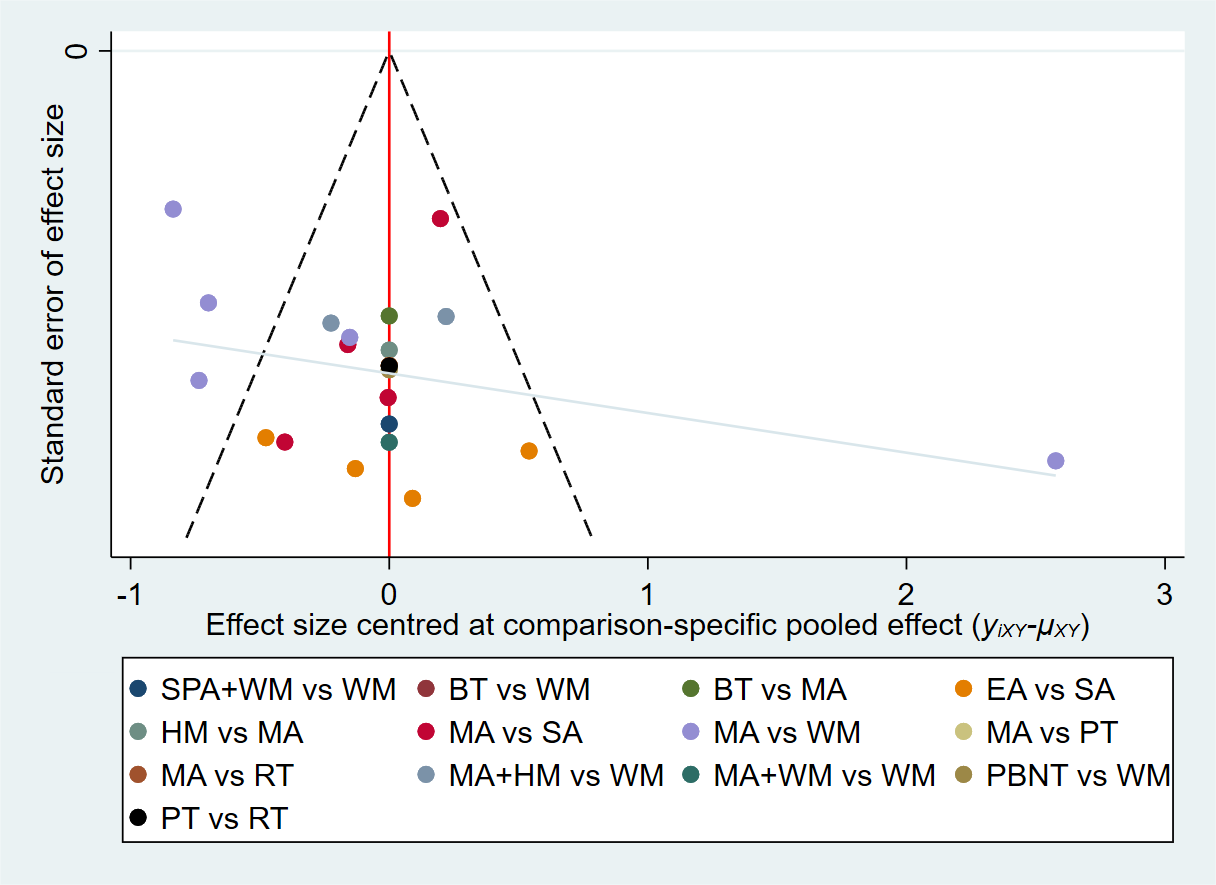
**

**Figure S3. Funnel plot of pain intensity**

**
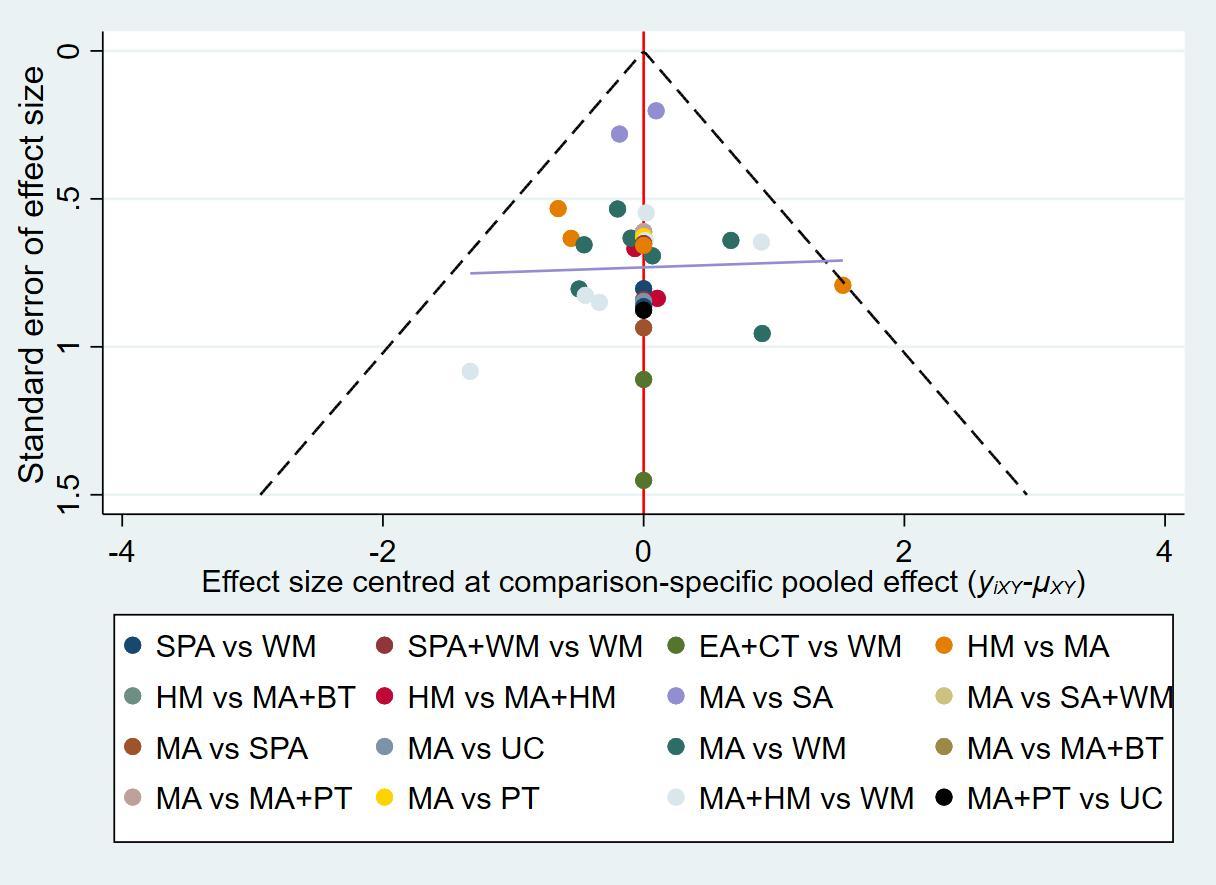
**

**Figure S4. Funnel plot of responder rate**
